# Faecal Pathogen Survival and Risks of Use of Ecological Sanitation By-Products in Burera District, Rwanda: A Quantitative Microbial Risks Assessment

**DOI:** 10.3390/ijerph23060816

**Published:** 2026-06-19

**Authors:** Celestin Banamwana, David Musoke, Theoneste Ntakirutimana, Esther Buregyeya, John Ssempebwa, Gakenia Wamuyu Maina, Charles Drago Kato, Lordrick Alinaitwe, Patrick Albert Ipola, Nazarius Mbona Tumwesigye

**Affiliations:** 1 Department of Environmental Health, College of Medicine and Health Sciences, University of Rwanda, Kigali P.O. Box 3286, Rwanda; tntakirutimana@nursph.org; 2 The Emerging and Re-Emerging of Infectious Diseases (TERID) Research Hub, College of Veterinary Medicine, Animal Resources and Bio-Security, Makerere University, Kampala P.O. Box 7072, Uganda; 3 Department of Disease Control and Environmental Health, School of Public Health, College of Health Sciences, Makerere University, Kampala P.O. Box 7072, Uganda; 4 Department of Community Health and Behavioural Sciences, School of Public Health, College of Health Sciences, Makerere University, Kampala P.O. Box 7072, Uganda; wamuyu_m@hotmail.com; 5 Department of Epidemiology and Biostatistics, School of Public Health, College of Health Sciences, Makerere University, Kampala P.O. Box 7072, Uganda

**Keywords:** Ecosan, excreta, faecal by-product, risk, microbe, sanitation

## Abstract

Reuse of human excreta and derivatives is becoming a common practice in areas with agricultural predominance. While in situ treated faeces through ecological sanitation (Ecosan), known as “faecal by-products” are being used to sustain soil nutrients and improve on-site sanitation, the concern remains about the health risks related to the survival of pathogens in these by-products in the community of farmers. This study assessed the survival of faecal pathogens and estimated microbial risks associated with the use of Ecosan faecal by-products in agriculture. The quantitative microbial risks assessment (QMRA) framework was used to estimate the risks posed by each faecal pathogen in solid and semi-solid faecal by-products under the probabilistic model of Monte Carlo simulation. *Ascaris lumbricoides* (6.5 eggs/gr), *Taenia* species (0.3 egg/gr), *Schistosoma* species (9.3 cercariae/gr), *Entamoeba* species (4.4 cysts/gr), and *Escherichia coli* (451 Cfu/gr) were detected in semi-solid faecal products. Exposure scenarios were observed throughout four critical points: vault faecal by-products removal/unloading, transport, collection, and application of faecal by-products in the gardens. Due to the presence of eggs and cysts, an estimated annual risk of infections was found in semi-solid faecal by-products with *Schistosoma* species (88%) and *Ascaris lumbricoides* (90%). Both concentrations were above World Health organisation (WHO) standards of associated infective risks of 0–10% of helminths in faecal sludge applied in the gardens. The users of faecal by-products, particularly farmers are exposed not only to high concentrations of helminth eggs but also to protozoa and bacteria with infective risks of *Entamoeba* species (99%) and *E. coli* species (62%). A stepwise implementation of faecal pathogens die-off during treatment of faecal by-products in compliance with the WHO’s 2018 guidelines can prevent the use of unsanitary faecal by-products. According to these findings, the proper control of intestinal protozoa and soil-transmitted helminths (STHs) should be enforced through personal protective measures in Burera district, Rwanda.

## 1. Introduction

Inadequate faecal treatment capacities and exposure to faecal products continue to induce microbial pollution in areas with the reuse of excreta resources [1]. The excreta derivatives, either from urine or faeces, named “urinal by-products or faecal by-products”, respectively, have been used for centuries in China and Vietnam for enhancing agricultural production [2]. Although the use of excreta became popular in these areas, an estimated burden of gastrointestinal infections up to 30% among farmers was recently reported in Vietnam [3,4]. Hence, faecal pathogens such as *Ascaris* and *Teania* species are most triggered by the use of excreta, and 530 million people are infected with *Ascaris* species in China [5,6]. About 10–25% of total illness in low- and middle-income countries is associated with the unbreakable lifecycle of faecal pathogens, and farmers were found to be the most vulnerable [7]. This is due to prolonged exposure to faecal pathogens during the excreta handling chain [8]. As such, this age-old practice has been improved due to the emerging excreta-treating technologies, including Ecological technology [9,10].

The faecal treatment through Ecological sanitation (Ecosan) technology indicated the difference in geographical patterns in which faecal pathogens die off [10,11]. It was noted that high annual temperatures (>30–40 °C) in Western African Countries accelerated the thermal drying of faecal products by removing water and sanitising the Faecal Sludge (FS) by accelerating the inactivation of pathogens [1,9]. Whereas in East African Countries (EAS), the faecal pathogens survived on Ecosan treatment due to the fluctuations in temperature, irregular use of ash, and high water content [12,13]. For example, in Malawi, the faecal by-products agro-practice favours existing microbial life cycle, particularly the most resistant *Ascaris lumbricoides* in the form of eggs [5,14,15]. This resulted in a high annual risk of ascariasis of 5.6 × 10^−1^ above World Health Organization (WHO) standards [16]. Such a discrepancy in annual risk estimates of these microbes in different geographical settings was observed. In addition, insufficiency of local data on microbial die-off and safety of faecal by-products informs further research in the areas where the use of faecal by-products was adopted. Although the safe Faecal Sludge Management (FSM) for manure has to fulfil Food and Agriculture Organization (FAO) and WHO standards [9,16,17], the health risks posed by on-site treated faecal matter in Ecosan have received less attention despite farmers exposure.

Studies [5,18,19] on health risks applying the Quantitative Microbial Risk Assessment (QMRA) model estimated the minimal risks of faecal sludge treated under diverse sanitation technologies, notably Faecal Treatment Plants (FTP). However, a higher risk estimate due to faecal by-products in agro-practice associated with resistant *Ascaris lumbricoides* in the form of eggs was observed in Malawi and Burkinafaso [20,21]. In contradicting or supporting treatment guidelines, specific and contextual studies applying sophisticated QMRA models in a wide range of variables are stressful and needed for risk estimation in farmers.

Other similar studies [22,23] evaluated the microbial risks using the risk matrix methods, and the risk rate of risks was biased as variability and uncertainty factors were not considered. Studies on faecal sludge using Quantitative Microbial Risks Assessment (QMRA) [5,19,22] were reliable in the actual identification of hazards, but are contextually bound on the assumptions.

In Rwanda, the Soil-Transmitted Helminths (STH) and Protozoa have remained among the top parasitic infections, with a prevalence of 62.7% in Burera district [24,25,26]. Consequently, the diarrhoea incidence rate associated with *Entamoeba* species remains high among children under five (16.5%) in Burera district [27,28,29]. In addition, *Ascaris* becomes a common pathogen in the community attending the health centres of Rugarama and Kirambo [30,31]. Although fresh faeces have undergone ash treatment and decomposition in a dry Ecosan system storage and decomposition, some faecal pathogens survive in different forms due to the poor use of ash and poor storage conditions. As a result, the faecal by-products remain poorly treated, and decomposition could take longer than expected. In that area, there is a local tendency for the anticipated use of faecal by-products among farmers due to the attractive advantages of higher crop yield [32]. In addition, farmers manipulate faecal by-products without the use of Personal Protective Equipment (PPE) during application in the gardens [33,34], which could induce multiple illnesses related to the locally prevalent faecal pathogens [29]. This study aimed to evaluate the survival of faecal pathogens in Ecosan faecal by-products using a QMRA framework that could estimate the risks associated with their use in agriculture to support the health risks management among farmers in Burera District, Rwanda

## 2. Materials and Methods

### 2.1. Study Area and Design

The study was carried out in Burera district, mostly in the predominant sub-entities of the district known as the sectors of Rugarama, Gahunga, and Cyanika. In these areas, under the promotion of Ecosan projects with the partnership of the local Government, 1000 Ecosan toilets were implemented in households and public places in the last decade. Subsequently, using Ecosan by-products (urine and faeces) as manure in the farmlands has become a common practice among farmers. Therefore, a cross-sectional study using the Quantitative Microbiological Risk Assessment (QMRA) framework was used to assess microbial risks associated with the use of Ecosan by-products among farmers.

### 2.2. Field Sampling

Samples were collected from a load of faecal by-products generated from households and public Ecosan in Burera district of Rwanda from January to March 2019, which was the period of intensive agricultural activities. At the time of disludge, samples were directly taken from the pile of faecal by-products by scooping up to 50 cm of deep at three levels: the top, middle, and bottom of the load, and then mixed in a single labelled bottle following the sampling procedure of Environmental Protection Agency (EPA) standards [35]. Based on the consistency of by-products, we adopted the classification model of stool consistency of the Bristol Stool Form Scale (BSFS) [8] where we aggregated seven types of stools’ consistency into two types, which are from one to four types as solid and from five to seven types as semi-solid faecal by-products.

**Figure 1:**
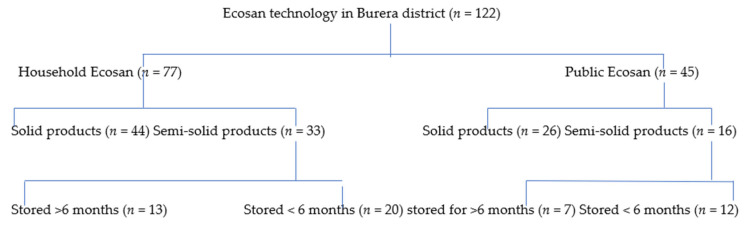
Sampling framework.

Samples were categorised into the solid matter with intact particles named “solid products” (*n* = 44), in contrast to slightly packed particles named “semi-solid products” (*n* = 13), stored for more than six months, and semi-solid products (*n* = 20) stored for less than six months from Ecosan vaults were collected at households. In addition, solid products (*n* = 26), semi-solid products (*n* = 7) stored for less than six months, and semi-solid products (*n* = 12), stored for less than six months from the vaults of Ecosan, were sampled in public places as indicated in Figure 1. Apart from bacteriological samples, other samples were kept in formalin solution and transported in cooler boxes to the microbiology laboratory of the University of Rwanda.

### 2.3. Quantitative Microbial Risks Assessment

**Figure 2:**
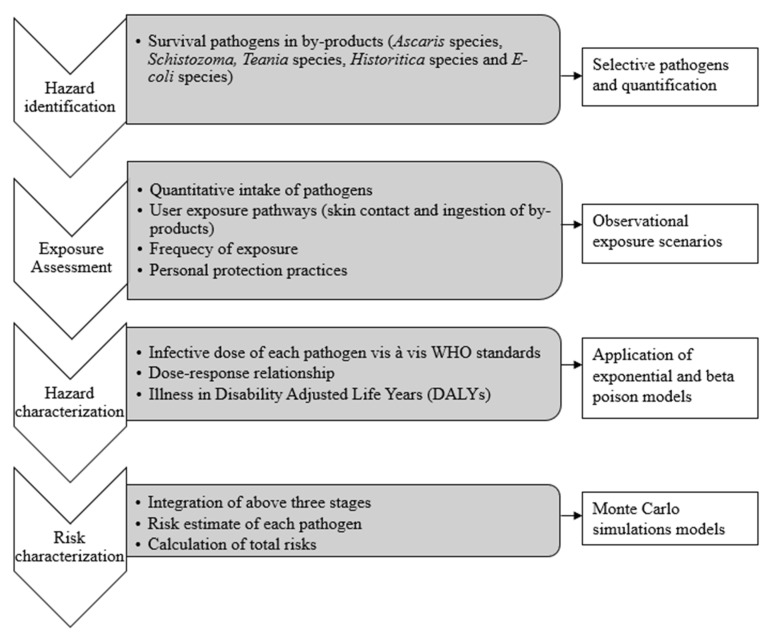
The steps of QMRA adopted by Boone et al. (2010) [18].

This analytical framework used in the study complies with four stages: hazard identification, exposure assessment, hazard characterisation, and risk characterisation, and the analysis follows each step and predicts infections associated with the users of faecal by-products (Figure 2).

#### 2.3.1. Hazard Identification

The identified hazards for the QMRA model are faecal transmitted route pathogens of high epidemiological findings reported in the previous literature [14,28,33,34]. This high disease burden is due to the long-time survival of these faecal pathogens in the environment, and hard to inactivate them with existing treatment options [1]. The identified faecal pathogens were *Ascaris lumbricoides, Schistosoma, Taenia*, and *Entamoeba* species, and common bacteria of *E. coli*. The selection of these faecal pathogens was informed by the previous studies on parasitic infections in Burera district [28,29,31].

The *Ascaris lumbricoides, Taenia*, and *Schistosoma* species were microscopically identified in an assay of one gram of faecal by-products following an adopted Formalin Ether Concentration Technique for intestinal worm parasites [35]. The cercariae form was extracted from the snails using the method of filtration and cercariometry, with visual microscopy at 500x. The visual identification of cysts of *Entamoeba* species was performed under microscopic magnification of 500×. *E. coli* was identified through the Membrane Filter Technique [36] and Chromocult Coliform/EC Agar after 24 ± 2 h of incubation then the selection of *E. coli* was identified on the media of Sorbitol-Mac Conkey (SMC) Agar.

The obtained results were recorded on an Excel spreadsheet. The data entry team reported any errors noticed during the data entry process to the principal investigator, which was minimised under a customised check programme built into the data entry. Data on targeted pathogens were calculated in Stata (v.14.2). Descriptive statistics with means and standard deviation showed the variation in the concentration of pathogens in faecal by-products from households and public Ecosan. Following the data obtained, further data on microbial risks of each pathogen was calculated following the mathematical models under the steps of exposure assessment, dose–response relationship, and risk characterisation.

#### 2.3.2. Exposure Assessment

Exposure to the faecal by-products was observed among farmers in the cooperatives, along with the manipulation of faecal by-products. The process started with the removal of faecal by-products from Ecosan vaults/unloading, transportation, collection, and application. The adopted observational checklist [22] was used to collect data on exposure scenarios along the manipulation process of faecal by-products, which means from the points of the removal of faecal by-products to their application in the garden [37]. In addition, such data were supported and completed by existing literature on epidemiological studies on similar occupational exposure in Malawi and Uganda [19]. According to previous epidemiological studies, the time of survival of STH and protozoa in faecal products was found to be more than 2 years [38,39]. This time was found to be longer compared to the bacteria, which can vary between 2 and 3 months under dry conditions [5]. The exposure pathways, such as skin contact and involuntary ingestion at each point, were identified with an estimate of exposure dose in six months of exposure during agricultural activities. These epidemiological data in similar study settings with the same agro-practices in use of Ecosan by-faecal products inform the actual study in Rwanda. In the areas where Ecosan was implemented with limited resources, complexities of adoption, such as flux of faecal parasites, lack of protective equipment, and knowledge gaps, were found to be crosscutting [3].

#### 2.3.3. Dose–Response Assessment

The infective dose of each investigated pathogen (*Ascaris lumbricoides, Taenia* species, *Schistosoma* species), Protozoa (*Entamoeba* species), and *E. coli* was determined in existing epidemiological studies performed elsewhere [1]. The daily exposure dose of each pathogen resulted in illness risk, which is commonly expressed as per person per day [40]. Given that the minimum dose of each pathogen was widely investigated in the most exposed populations in similar study areas in Malawi [5] and Uganda [1], some exposure parametric variables were taken into account to adjust such existing dose–response models to the best fit of the actual scenarios. Therefore, the concentration of each pathogen (number of ingested pathogens) and annual exposure associated with infectivity were extrapolated to reduce uncertainty in the model, considering the WHO/FAO guide on the use of faecal sludge in crop production [18,41].

Since the interaction of one pathogen in the host can induce disease, *Ascaris, Taenia, Schistosoma*, and *Entamoeba* species follow the exponential model [39]. The host interaction of each pathogen follows the exponential model within time, and therefore, the ingested dose by days in faecal by-products was calculated following the formula:

[Nt] = No × e^−kt^ (1)

“Nt” = the concentration of eggs or cysts at a certain time “t” (day), “No” = the concentration of the eggs or cysts per gram of the by-product, “e” = an exponential value = 2.6, “k” = a constant on the decay of pathogens varies to 0.005 for eggs of STH and 0.019 for cysts of *Entamoeba* species [42].

It is not evident to get sick when cross-contact with one pathogen of *E. coli*. There is a threshold of pathogens and host immunity that can trigger a disease [39], which follows the Beta-Poisson model [5,39,40] as indicated by the equations below. It was noted that the infective dose of *E. coli* follows the beta-Poisson model since its infectivity requires a certain dose of pathogens in interaction with a host.

P_Inf_ = 1 − {1 + (*d*/*β*)}^−*α*^ (2)

where “P_inf_” = the likelihood of infection, “*d*” = the estimated ingested dose, “*α*” = a constant for each faecal pathogen, stands for the dose–response. The dose–response parameters used for *E. coli* species “*N*_50_” = 1120 and “*α*” = 0.2099 [9].

#### 2.3.4. Risk Characterisation

This step was an integral part of the above three cited steps of QMRA, and the estimated risk of each parasitic infection in faecal by-products was calculated by exponential and Beta Poisson models as cited below.

PA = 1 − (1 − P_inf_)^n^ (3)

“P_inf_” = risk of infection given by a single exposure event and “n” = the frequency of exposure per year [39]. A comprehensive picture of the microbial risks of each pathogen was estimated by minimising variability and uncertainty. An assimilation method was applied under probabilistic models of Monte-Carlo simulations with 10,000 trials to estimate the risks posed by each pathogen. Since farmers were frequently exposed to the infective products at different critical points during exposure scenarios, multiple assimilations for exposure dose were adopted with the probability of zero to one [11]. Given that, multiple exposures occur inconstantly, the response differs from one person to another, and in case of no die-off of pathogens, a risk simulation was plotted to minimise such parametric variabilities with an estimate of median risks following MATLAB (version 5.0) [43] in compliance with the assumptions considered during the established WHO (2006) guidelines.

## 3. Results

### 3.1. Identification of Pathogens in Faecal By-Products

The cysts of *Entamoeba* species were found in the faecal by-products, and *E. coli* was detected in semi-solid products. Pathogens such as *Ascaris lumbricoides, Taenia* species, *Schistosoma, Entamoeba* species, and *E-coli* were detected in 1 g of faecal by-products. Table 1 indicates the statistical significance of the difference in STH, protozoa, and bacteria concentrations between Ecosan in households and public places. Such differences in mean concentration of pathogens followed the visual consistency of the faecal by-products. The concentration of *A. lumbricoides* in semi-solid faecal by-products was 4.8 eggs per gram at household Ecosan, which was doubled in similar products at public Ecosan. The statistical correlation was found with a concentration of *Entamoeba* species (2.5 cysts/gr) at household versus (5.5 cysts/gr) in the same semi-faecal by-product at Public Ecosan.

The faecal pathogen concentration was also determined in solid and semi-solid faecal by-products after being removed from the Ecosan vaults (Table 2). There was a higher concentration of each pathogen in semi-solid faecal by-products than in solid faecal by-products. It was found that *Ascaris lumbricoides* (6.5 eggs/gr), *Schistosoma* species, and *Entamoeba* species in semi-solid products (4.4 cysts/gr) were almost double compared to the same solid faecal by-products. A high concentration of *E-coli* (451.9 Cfu/gr) was detected in semi-solid faecal by-products, whereas the same pathogen was (111.7 Cfu/gr) in solid faecal by-products.

### 3.2. Exposure Assessment

**Figure 3:**
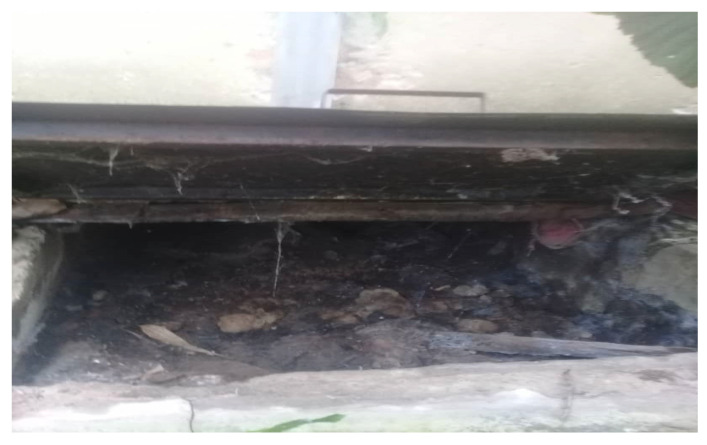
The faecal by-products stored in Ecosan vault.

The key critical points in handling scenarios of by-products started from vaults faecal by-products removal (Figure 3), loading zone, transport, and application of faecal by-products in the gardens. Given that, the process of manipulation of faecal by-products for agriculture involved only the same farmers, an estimated exposure dose of identified faecal pathogens through multiple transmission pathways at critical points was estimated (Table 3). During the field visits, spades were not available on-site; people used hoes for faecal vault emptying and touched faecal by-products with their hands with close contact. In addition, they also stepped inside the faecal vaults without wearing gloves and boots. At the point of transport of faecal by-products, people unloaded the faecal by-products into sacks, due to a lack of wheelbarrows, and carried them on from Ecosan vaults to the collection zone, up to their gardens. In the same scenario of transport, there was hand touching of faecal by-products, which end up in the mouth and are ingested. At the point of the collection zone, there was a spread of faecal by-products by dogs and chickens reared in the surrounding environment, which ends up contaminating the vicinity vegetable garden. At the point of application of faecal by-products in the gardens, it was observable that the farmers measured 20 kg of faecal by-products in sacs to spread on the field using their hands without gloves. A transfer of faecal by-products from the hands to the mouth during the faecal by-products emptying scenario was estimated as the main ingestion route of *E-coli* and *Ascaris lambricoids*, Entamoeba species. In this study, an assumption was made that the faecal by-products quantity ingested or dermal contact led to the worst-case scenario of multiple infections, as assumed in the previous studies [5]. A study performed in Malawi [40], the adults unintentionally ingested between 0.03 and 0.1 g per day, while dermal contact was estimated to be 0.3 g per day under no protective circumstances [37]. As the same farmers are more exposed to these faecal by-products for three months in the two agricultural seasons of the year, it means that an estimated exposure dose in grams was estimated for six months in the year following the formula below [5].

EDPY = EEDPD × ETD × ETW × ETM(4)

“EEDPY”: Estimated Exposure Dose of faecal by-products per person by year, “EEDPD”: Estimated exposure dose per person per day; “ETD” = Exposure Time in Days per week; “ETW” = Exposure Time in Weeks per month; “ETM” = Exposure Time in Months per year. According to Equation (1), this implied the annual exposure dose of 7.2 g of faecal by-products ingested with an assumption of average ingestion of 0.05 g a day and 0.15 g with dermal contact, equivalent to 21.7 g of faecal by-products intake/day/year [37]. Since the concentration of faecal pathogens in one gram is known, it is assumed that the estimated dose of faecal pathogens by a person in a year is indicated in Table 3.

### 3.3. Dose–Response Assessment

The infective dose was documented from the literature on identified parasitic pathogens and epidemiological data. The dose–response curves were used to determine if there was a relationship between the concentrations of pathogens ingested in a certain volume of faecal by-products and the likelihood of getting an infection. The mathematical model used to determine the dose and pathogen effects was identified and varied according to the type of pathogens. An exponential model was used to calculate the infective dose of *Ascaris, Lumbricoides, Taenia* species, and *Schistosoma* species, whereas *E. coli- 0157:H7* and *Entamoeba* species were calculated by using a beta poisson model, as indicated in Table 4.

### 3.4. Risk Characterisation

The parasitic infection burden counted by person per year combined all five cited pathogens. The probability of illness, along with the obtained concentration of each pathogen at each exposure point, was calculated. Data were adjusted from the literature to estimate personal exposure quantity and frequency. The high-risk estimate of infection of *Schistosoma* species (cercariae was found to be 980 × 10^−1^ equal to 98% in semi-solid faecal by-products, whereas such risks decreased to 360 × 10^−1^ equal to 36% in solid faecal by-products. In addition, *Entamoeba* species (*Cysts*) presented a higher risk of infection, 290 × 10^−1^ equivalent to (29%) in semi-solid faecal by-products (Table 5).

## 4. Discussion

In the framework of understanding the health risks posed by the use of Ecosan faecal by-products in agriculture, a Quantitative Microbial Risk Analysis (QMRA) Model was applied to reduce the uncertainty of the estimation of risk. Combined with the exposure to local scenarios (faecal by-products removal/unload, transport, collection, and application), survival faecal pathogens were identified and their infective dose was documented. The infective dose of each pathogen in both solid and semi-solid faecal by-products was calculated and was far above WHO standards.

The presence of eggs and cysts contributed to an estimated annual risk of infections in semi-solid faecal by-products with *Schistosoma* species (9.9 × 10^−1^) and *Entamoeba* species (9.9 × 10^−1^). Hence, such a risk estimate was above the WHO standards of 10^−4^ pppy of the use of faecal sludge in gardens [5]. The study provides insights into the solid treatment of human excreta under Ecosan and the infectious risks of the use of faecal by-products in the area, with 62.7% of infections related to pathogens cited above in Burera district, Rwanda.

A discrepancy between the design of Ecosan technology and field implementation resulted in the high survival rate of faecal pathogens. Studies [46] performed on Vietnamese Ecosan latrines stated that the irregular practical use of ash additives slows down the pathogen die-off despite having a robust technological design. Such findings correlate with the visual consistency of semi-solid faecal by-products due to the poor use of ash. Another study [47] found the local imitation of Ecosan technology, particularly UDDT, by inserting a urine-diverting squatting pan in wood slabs, which makes cleaning surroundings difficult. Diverse research on Ecosan has reported 100% removal of pathogens throughout the composition process [48,49]. Although Ecosan has been used for many years in diverse societies, users are unlikely to meet the operational requirements [50]. Therefore, such technology itself does not provide a guarantee of complete removal of faecal pathogens without involving users’ practices. While handling the process of the faecal by-products, the reality in areas with limited resources, including Rwanda, met difficulties in the use of Ecosan [47,51]. Thus, the faecal by-products remain critically safe regardless of the design of Ecosan technology. Studies on Faecal Sludge Management (FSM) have applied the QMRA model with an advance in microbial die-off quantification techniques [18,24,52]. However, the standard practical options for proper maintenance are not commonly executed for reliable and accurate emerging sanitation technologies, including Ecosan technology, which renders uncertainties of use of faecal by-products, and hence health risks among users [53].

*Ascaris lumbricoides, Schistosoma* species, and *Taenia* eggs were the STHs found in Ecosan faecal by-products. In addition, *Entamoeba* species, and *E. coli-0157:H7* were all detected from household and public Ecosan faecal by-products. A high concentration of these faecal pathogens was found particularly in semi-dry faecal by-products from public Ecosan, which exceed the values established by WHO standards of a maximum of 1 Nematode egg/L in treated faecal by-products to be used in the gardens [42]. Such a high concentration of these pathogens in faecal by-products reflects the existing high prevalence of each and the inefficiency of on-site treatment through Ecosan. Similar study findings on high STHs in Malawi and South Africa were also evidence of poor treatment of faecal sludge [5,39]. Eggs of *Ascaris lumbricoides* can survive against solid excreta treatment and decomposition, which later leads to the high risks of epidemiological infections of *Ascaris* species. An evident estimated risk of *Ascaris* infections was found to be higher in semi-solid faecal by-products than in solid faecal by-products. This can be attributed to the early harvest of incomplete decomposition of faecal by-products stored in Ecosan vaults for less than six months and irregular use of ash additives as stipulated in the WHO/FAO guide [54], so as to safely the faecal by-products and safeguard the public health of users.

The concentration of cercariae form of *Schistosoma* in *snails* was found to be higher in semi-solid faecal by-products from public Ecosan, which is related to the high prevalence (>50%) of Schistosomiasis in the community vicinity of lakes Burera and Ruhondo, located in Burera district. Although the by-product decomposition under a solid system of Ecosan occurred with conditions (ash additives, time of storage, deprivation of oxygen, and water), the high concentration of cercariae from *Schistosoma* in snails in semi-solid faecal by-products remains unpredictable due to the high water content in the faecal by-products. The high accumulation of cercariae forms in snails was found in faecal sludge in Senegal [39], and their removal rate was due to the dry treatment options and time of decomposition that can be extended to 8 to 12 months [55]. However, the use of semi-solid faecal by-products was evidence of the early harvest of faecal by-products, which contain water, and depends on the local farmers’ demands. There was a tendency for actual exposure to the pathogens through different critical pathways. Along critical exposure points, from the by-product removal to their application on the farms, farmers wear neither gloves nor shoes, and frequently touch their mouths intentionally without hand washing. Similar practices were observed in similar resource settings of Malawi [5], but differ from the categories of exposed farmers who are the most vulnerable to the high dose ingested. Therefore, proper combined methods of health-protective measures among farmers must be carefully considered before being engaged in faecal by-products practice.

An estimated high risk of infection of amebiasis in the use of faecal by-products in the gardens was associated with a high concentration of cysts of *Entamoeba* species in the same faecal by-products, above the WHO/FAO values of two cysts per gram of sludge usable in agriculture. In addition, the observed local protective measures were critical as farmers handled the faecal by-products at different critical points without using either gloves or any other protective materials. This high-risk rate, similar to previous studies [56,57], was also confirmed in epidemiological studies on risk factors of amebiasis in endemic areas [31,56,58]. By contrast, a lack of evidence of an association between the risk of *Entamoeba* infections and the use of excreta in a case–control study in Vietnam [57] was due to the study’s limited exposure scenarios. The higher concentration of cysts of *Entamoeba* in public Ecosan faecal by-products was due to high enteric *Entamoeba* species infected travellers who have used such Ecosan. According to the data collected on *Entamoeba* species in Vietnam, tourists from developing areas transmit such pathogens through the use of the public latrines [37]. An outcome of a high prevalence of amoebiasis in the Vietnamese farmers’ users of faecal sludge was reported [59]. There is a need to set guidelines on the proper use of faecal products, including microbial assessment before application in the gardens, to protect community health.

The colony-forming units (Cfus) of *E. coli-0157:H7* in both solid and semi-solid faecal products were higher than the WHO standards of 1000 organisms per gram of sludge. The only difference was about four times the concentrations of the same pathogen in semi-solid faecal products. According to studies [19,60,61], assessing the process of faecal sludge decomposition, above 36 °C, thermotolerant coliforms are reduced from 10^5^ Cfu/g to the detection level of 10 Cfu/g as indicators of the absence of other pathogens in faecal material. However, compared to similar studies in Vietnam [62] and China [40], there was no conformity of results from these different countries. Such a discrepancy in findings can be attributed to variations in geographic environmental conditions during the time of decomposition of excreta. In addition, some faecal coliforms can survive dry treatment, such as *E. coli-0157:H7* [62]. Study findings on annual risk estimates of the most parasitic pathogens in Rwanda came to fill existing gaps of local data on faecal by-products for further treatment and users’ protection against cross-contamination of faecal pathogens. This can eradicate the transmission of such bacteria and other related faecal pathogens for reducing the incidence of diarrhoea among the community users of the faecal by-products.

This is the QMRA study model, built on a series of evidence that supports the prediction of risks of diverse infections in an endemic limited service of excreta management area. In this context, farmers were vulnerable as common users of faecal by-products on the farms. The study used laboratory-based findings combined with systematic field information and literature to estimate the risks of infection. Such epidemiological models are used to calculate and minimise the uncertainty and variability to avoid biased result estimates. The study model is built on predictive assumptions of exposure scenarios and individual susceptibility, tested outside the area of study, such as Malawi and Uganda, which can induce errors in the estimation of risks of infections. The high-risk estimate was induced by worst-case exposure scenarios. However, such uncertainty was minimised under consideration of similar study findings on actual exposure to Ecosan by-faecal products among farmers in Sub-Saharan Countries. In these countries, such vulnerable people are experiencing similar complexities of adoption of Ecosan, including inadequate protective measures, sanitation shortfalls due to financial constraints, and knowledge gaps of the use of Ecosan by-products. It is also limited to the number of study area predominant faecal pathogens, which can survive in the form of eggs, cercariae, or cysts in environmental stress. Establishing local decay rates (k values) for STHs and protozoa under Burera district’s specific environmental conditions can enhance the safe agro-practice among farmers in Rwanda.

## 5. Conclusions

There is a high risk of infections related to *Ascaris, Lumbricoides, Taenia* species, *Schistosoma* species, *E. coli-0157:H7,* and *Entamoeba* species in the use of faecal by-products, particularly the semi-solid faecal by-products generated from household and public Ecosan. A high-risk estimate found by applying a QMRA framework was evidence of improper use of the WHO/FAO guidelines for the use of faecal sludge in agriculture in such endemic areas with limited resources. As such, the practice of the use of faecal by-products is widely adopted; these guidelines continue to be seen as strangers in limited resource areas where farmers are mostly attracted by agricultural returns. Regular monitoring is crucial in the control of faecal pathogens’ die-off during the decomposition before removal from Ecosan vaults. Uncertainty regarding the inactivation of faecal pathogens has to be stressful to reduce the associated health risks, either by improving treatment with ash or prolonged storage. While manipulating the faecal by-products, there is a need to enforce and implement specific preventive measures, such as wearing gloves, facemasks, and boots, and hand washing after gardening activities. It is therefore vital for public health actors to implement strict guidelines of on-site dry sanitation for Ecosan in the reduction of the faecal pathogens in faecal by-products for sustainable use in agriculture in Burera district, Rwanda.

## Figures and Tables

**Table 1:** The mean concentration of pathogens in faecal by-products in households and public.

Household Ecosan					Public Ecosan			
Pathogens	Solid Faecal By-Products		Semi-Solid Faecal By-Products		Solid Faecal By-Products		Semi-Solid Faecal By-Products	
	Mean (Std)	95% CI	Mean (Std)	95% CI	Mean (Std)	95% CI	Mean (Std)	95% CI
STHs (eggs per gr)								
*Ascaris lambricoides*	2.3 (0.2)	1.7–2.6	4.8 (0.3)	4.2–5.5	4.2 (0.6)	2.9–5.5	9.8 (0.8)	8.1–11
*Taenia* Species	0.1 (0.01)	0.1–0.1	0.3 (0.03)	0.2–0.9	0.3 (0.02)	0.2–0.3	0.5 (0.07)	0.3–0.6
*Schistosoma specie* (cercariae)	0.6 (0.2)	0.2–1.0	8.8 (0.9)	6.9–10.7	3.2 (0.8)	1.4–5.0	10.2 (1.4)	7.4–13.1
Protozoa (cysts per gr)								
*Antamoeba* species	2.5 (0.3)	1.9–3.2	3.9 (0.3)	3.2–4.5	3.5 (0.3)	2.8–4.3	5.5 (0.36)	4.8–6.2
Bacteria (Cfu/gr)								
*E-coli*	90.1 (20.2)	49.8–130.3	407.4 (53.6)	300.6–514.2	144.1 (12.5)	118.8–169.4	540.8 (77.6)	384.4–697.3

**Table 2:** The mean average of pathogens in solid and semi-solid faecal by-products.

Pathogens	Solid Faecal By-Products		Semi-Solid Faecal By-Products	
	Mean (Std)	95% CI	Mean (Std)	95% CI
STHs (eggs per gr)				
*Ascaris lambricoides*	3.0 (0.3)	2.4–3.6	6.5 (0.4)	5.6–7.4
*Taenia* Species	0.2 (0.01)	0.1–0.2	0.3 (0.03)	0.3–0.4
*Schistosoma* species (cercariae)	1.6 (0.4)	0.8–2.4	9.3 (0.7)	7.7–10.8
Protozoa (cysts per gr)				
*Antamoeba* species	2.9 (0.2)	2.4–3.4	4.4 (0.2)	3.9–5.0
Bacteria (CFUs per gr)				
*E-coli*	111.7 (13.4)	85.1–138.3	451.9 (44.5)	363.7–540

**Table 3:** Estimate intake of faecal by-products and faecal pathogens along with exposure scenario.

Pathogens	Exposure Pathways		Solid Faecal By-products	Semi-Solid Faecal By-Products
Scenario		Mean Faecal by-Products Dose Absorbed (g/Day/Year)	Mean Pathogen Dose by Person/Day/Year	Mean Pathogen Dose by Person/Day/Year
STHs (eggs per gr)				
*Ascaris lambricoides*	Ingestion	7.2	21.6	46.8
*Taenia* Species	Ingestion	7.2	1.4	2.1
*Schistosoma* species (cercariae)	Dermal contact	21.7	34.7	201.8
Protozoa (cysts per gr)				
*Antamoeba* species	Ingestion	7.2	11.5	31.6
Bacteria (CFUs/gr)				
*E-coli*	Ingestion	7.2	804.2	3253.6

**Table 4:** Faecal pathogens dose–response.

Mean Pathogen Dose by Person/Day/Year (p/d/y)	Infective Dose		Response Levels		Infective Dose	Model	Source
Scenario	Solid Faecal By-Products	Semi-Solid Faecal By-Products	Solid Faecal by-Products	Semi-Solid Faecal By-Products	WHO/FAO Values	-	-
*A. lumbricoides*	3.0	6.5	0.95	0.99	10^0^ to 10^1^	Exponential model	[19]
*Taenia* species	0.2	0.3	0.19	0.26	10^0^ to 10^1^	Exponential model	[44]
*Schistosoma* species	1.6	9 × 10	0.80	1.0	10^0^ to 10^1^	Exponential model	[44]
*Entamoeba* species	2.9	4.4	0.94	1.0	10^0^ to10^1^	Exponential model	[44]
*E. coli*	11 × 10	45 × 10	0.16	0.62	10 to 10^2^	Beta-Poisson model	[45]

**Table 5:** The total estimate of risks due to exposure to faecal by-products.

Solid Faecal By-Products					Semi-Solid Faecal By-Products		
Faecal Pathogen	Route of Exposure	Annual Exposure Faecal Dose (gr)/Person	Annual Exposure to Pathogen Dose/Person	Total Risks Estimate (pppy)	Annual Exposure Faecal Dose (gr)/Person	Annual Exposure to Pathogen Dose/Person	Total Risks Estimate (pppy)
*A. lumbricoides* (egg)	Ingestion	7.2	21.6	9.5 × 10^−1^	7.2	46.8	9.9 × 10^−1^
*Taenia* species *(eggs)*	Ingestion	7.2	1.4	1.9 × 10^−1^	7.2	2.1	2.6 × 10^−1^
*Schistosoma* species(cercariae)	Ingestion Dermal contact	21.7	34.7	8 × 10^−1^	21.7	20 × 10	1 × 10^0^
*Antamoeba* species *(Cysts)*	Ingestion	7.2	11.5	9.4 × 10^−1^	7.2	31.6	9.9 × 10^−1^
*E. coli* *(Cfu)*	Ingestion	7.2	80 × 10	1.6 × 10^−1^	27.2	32 × 10^2^	6.2 × 10^−1^

Cfu: Colony-forming units, Gr: Gram, pppy: per person per year.

## Data Availability

All data presented in this study are within the manuscript.
